# Supplementary figures and images for: Evaluation of Neurotropic Activity and Molecular Docking Study of New Derivatives of pyrano[4″,3″:4′,5′]pyrido[3′,2′:4,5]thieno[3,2-d]pyrimidines on the Basis of pyrano[3,4-c]pyridines
Source: Molecules. 2022 May 24;27(11):3380. doi: 10.3390/molecules27113380 (PMC9182472; doi:10.3390/molecules27113380)

Compound 3a

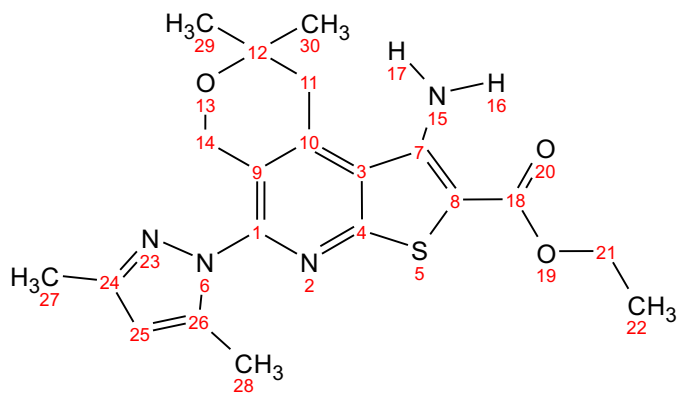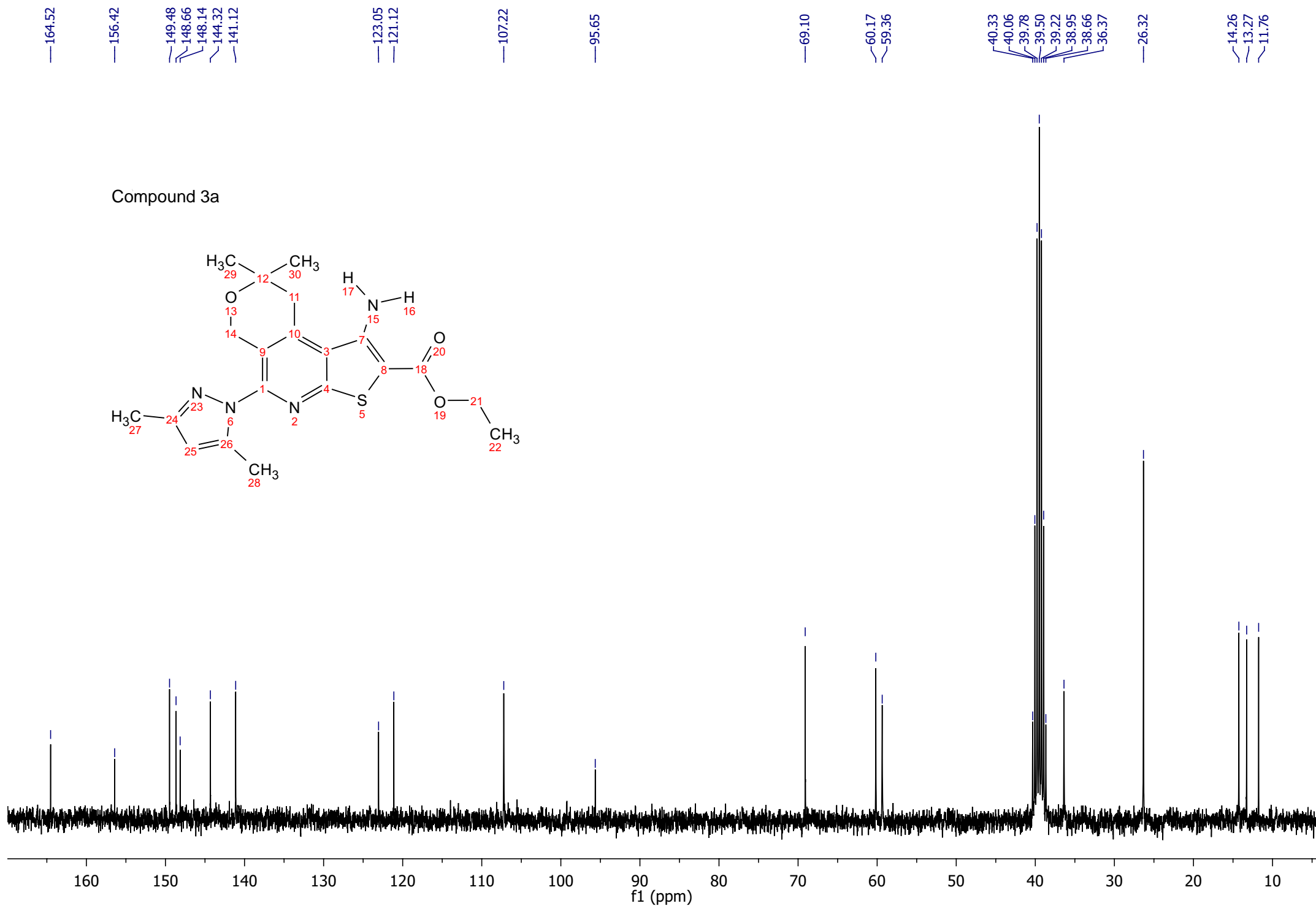

Supplement: Supplementary file 1 [file molecules-27-03380-s001.zip › comp.3a_C13.pdf]

Compound 3a

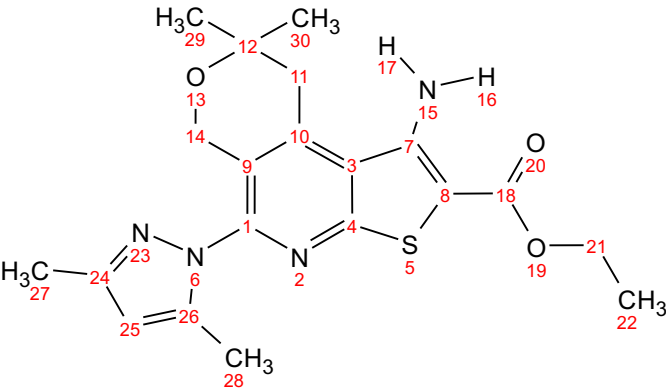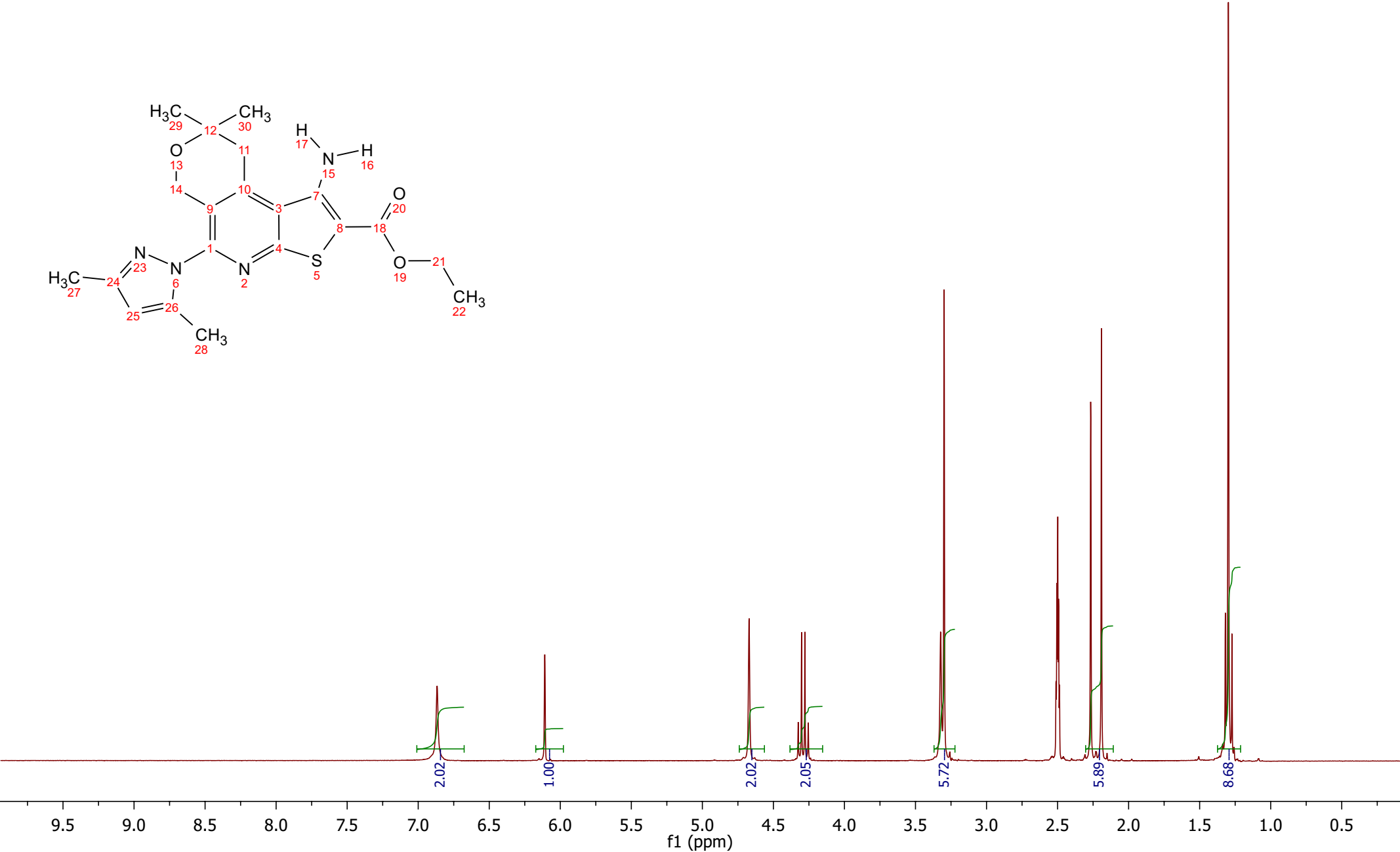

Supplement: Supplementary file 1 [file molecules-27-03380-s001.zip › comp.3a_H1.pdf]

Compound 3b

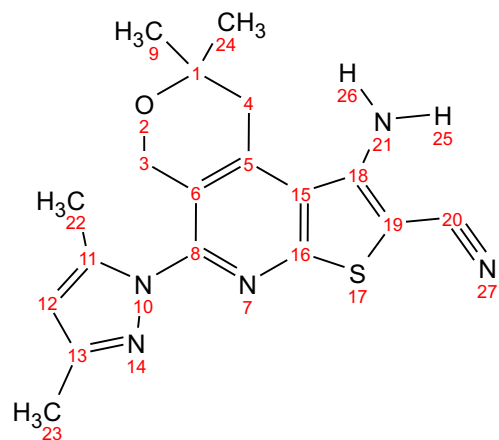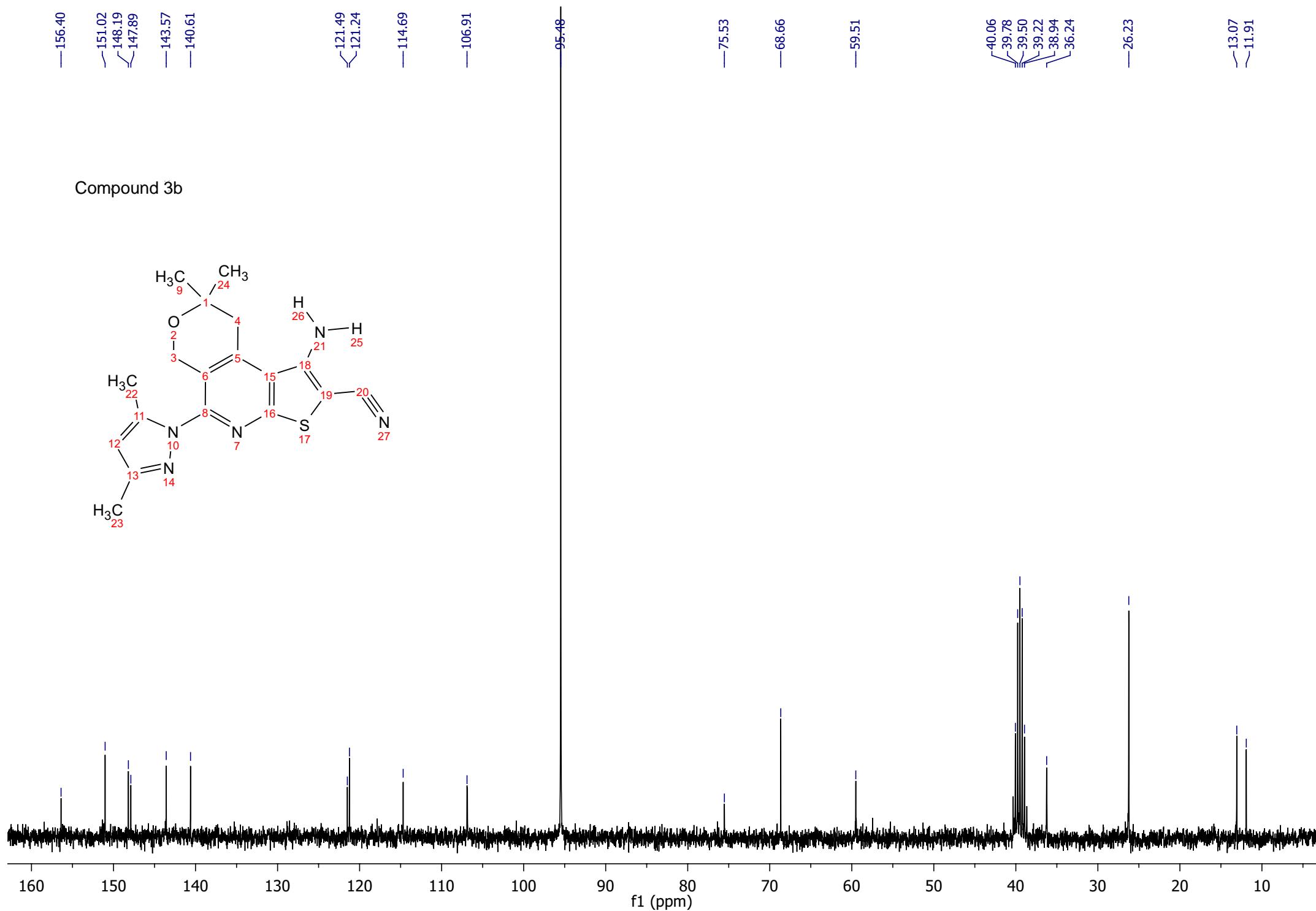

Supplement: Supplementary file 1 [file molecules-27-03380-s001.zip › comp.3b_C13.pdf]

Compound 3b

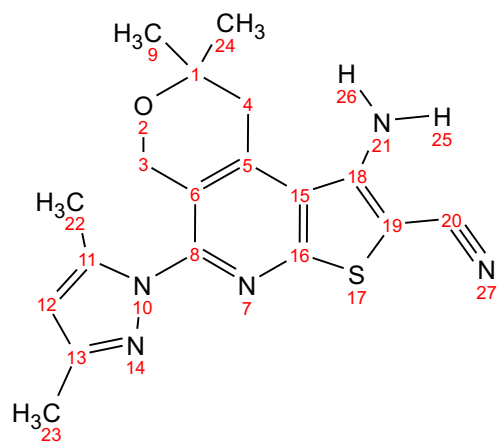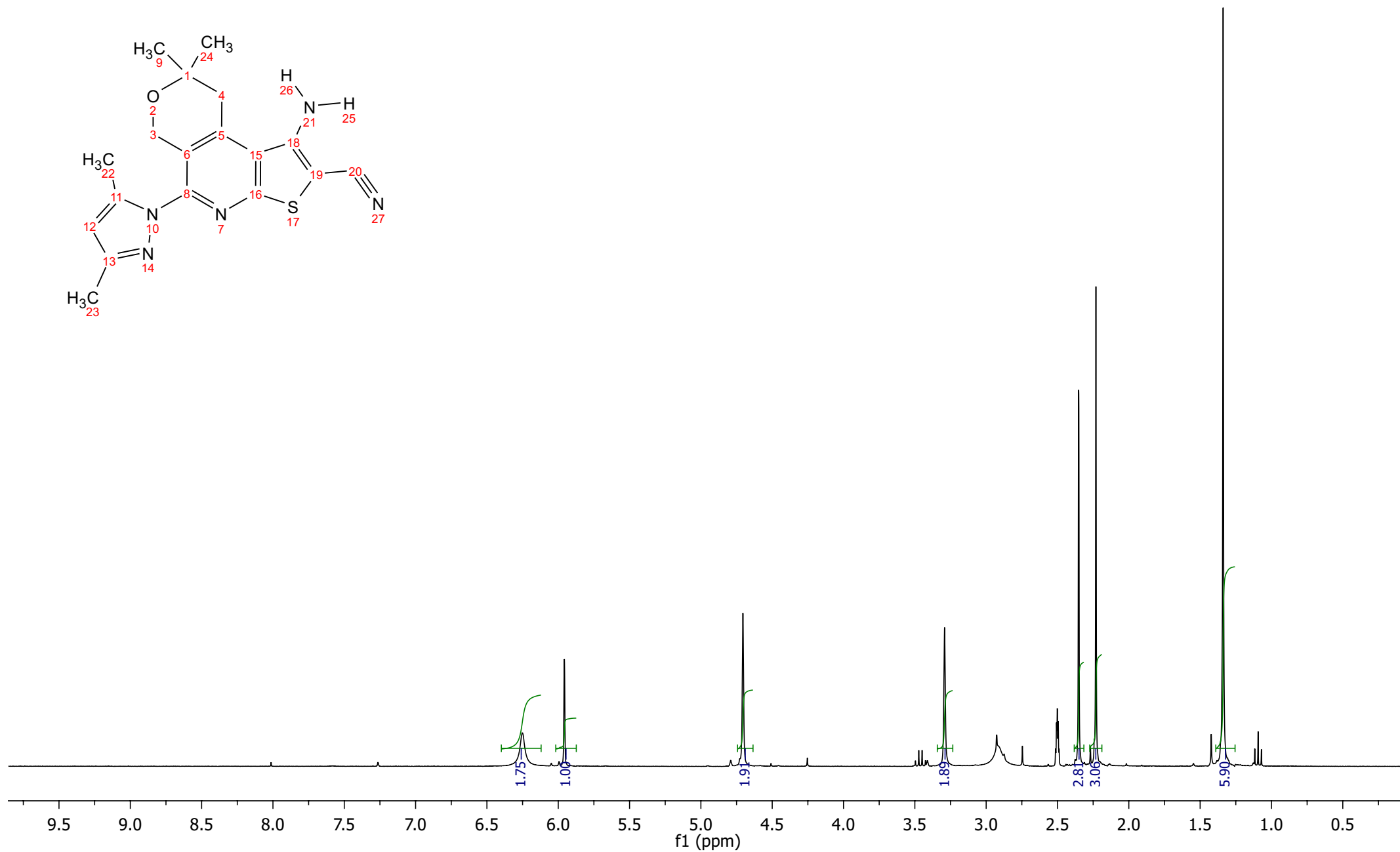

Supplement: Supplementary file 1 [file molecules-27-03380-s001.zip › comp.3b_H1.pdf]

Compound 3c

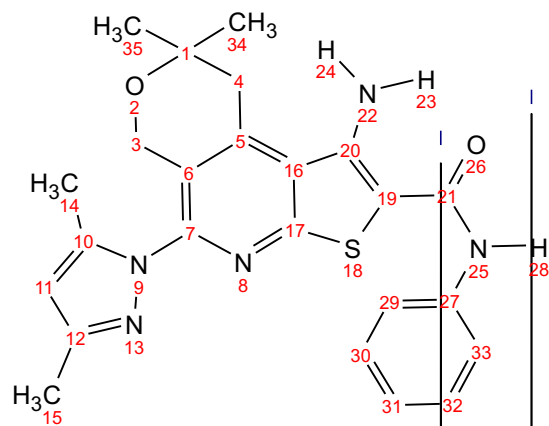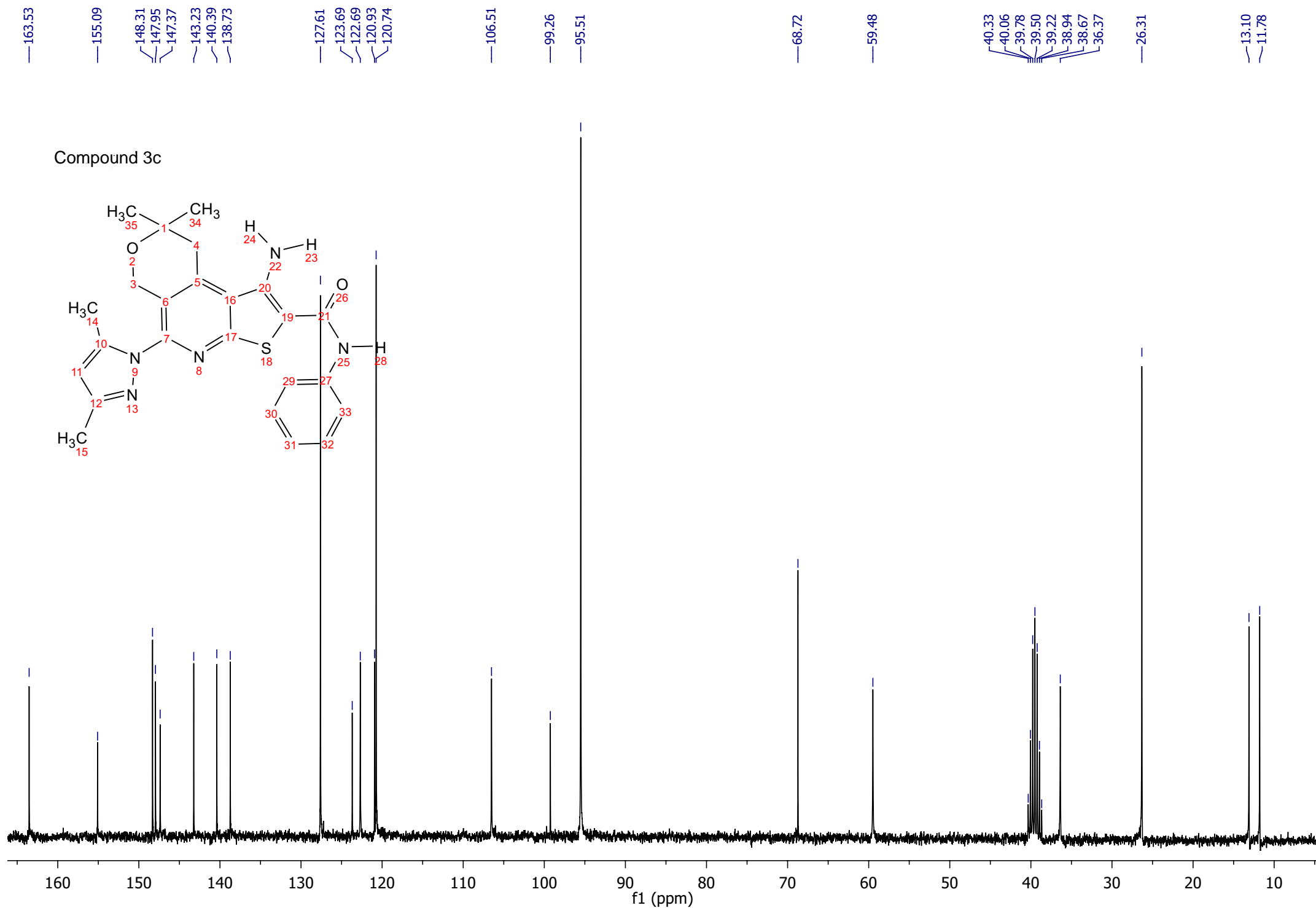

Supplement: Supplementary file 1 [file molecules-27-03380-s001.zip › comp.3c_C13.pdf]

Compound 3c

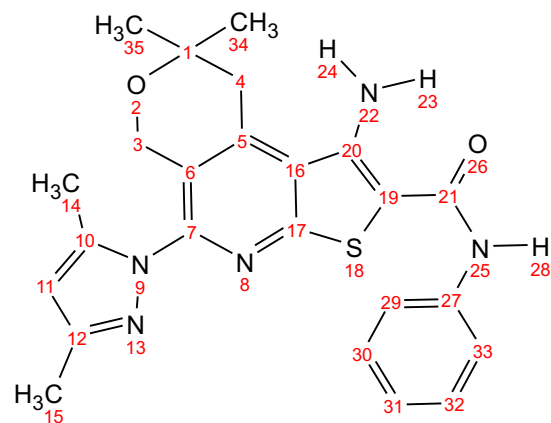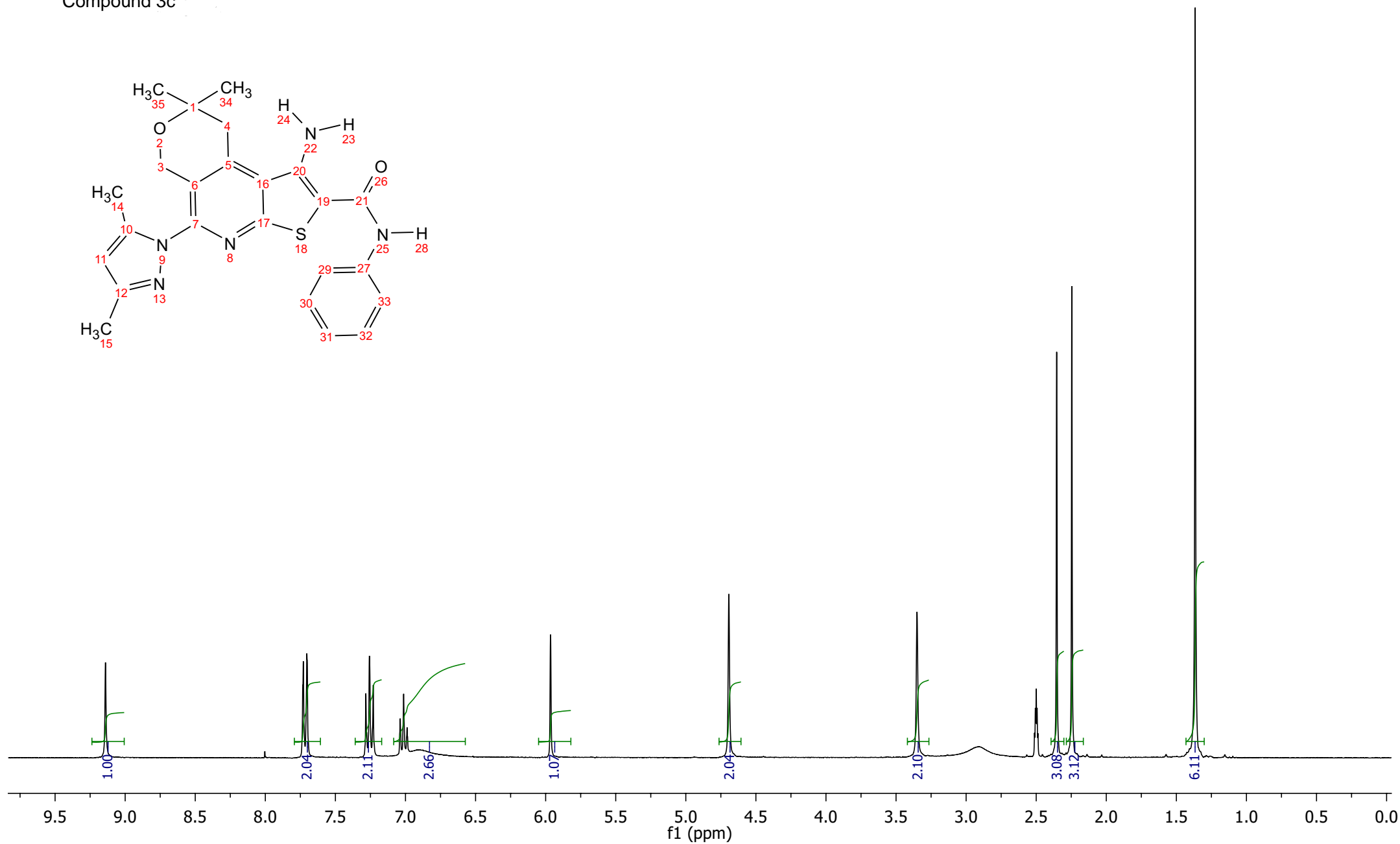

Supplement: Supplementary file 1 [file molecules-27-03380-s001.zip › comp.3c_H1.pdf]

Compound 3d

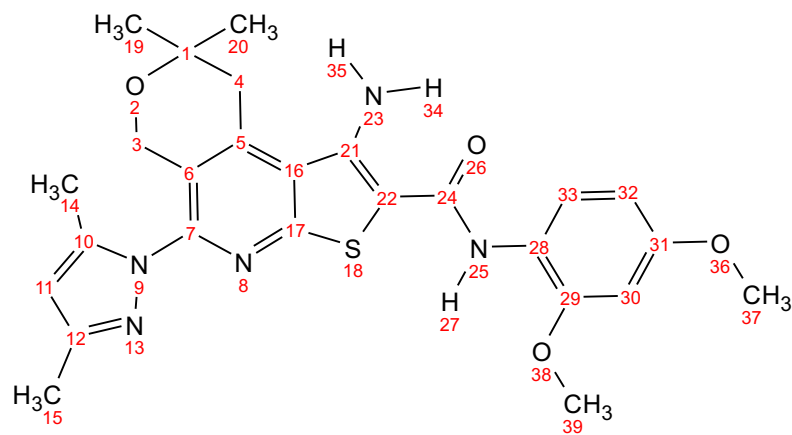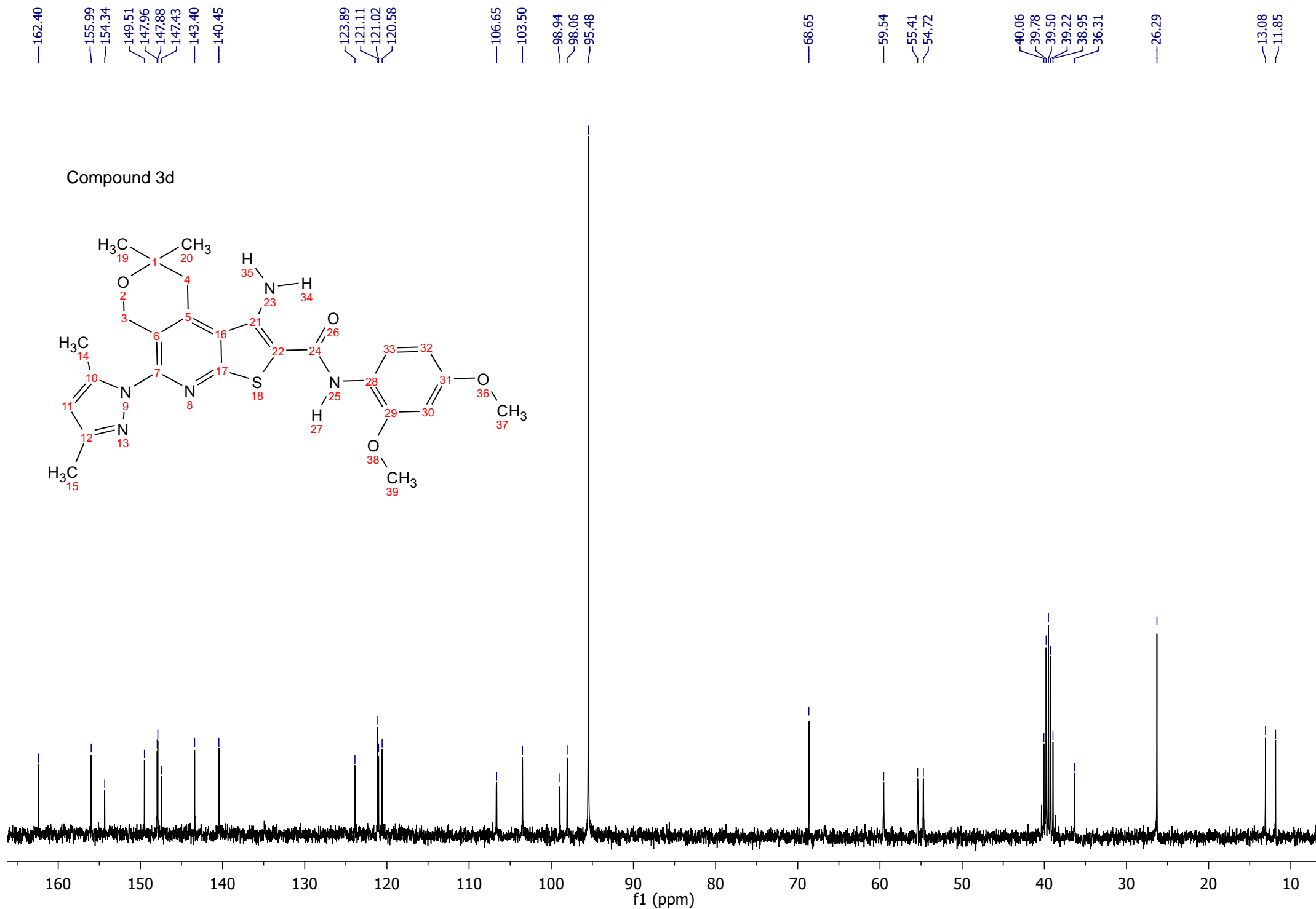

Supplement: Supplementary file 1 [file molecules-27-03380-s001.zip › comp.3d_C13.pdf]

Compound 3d

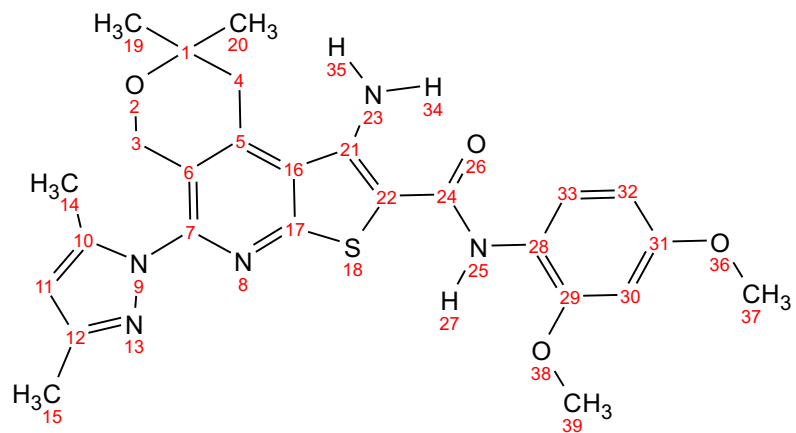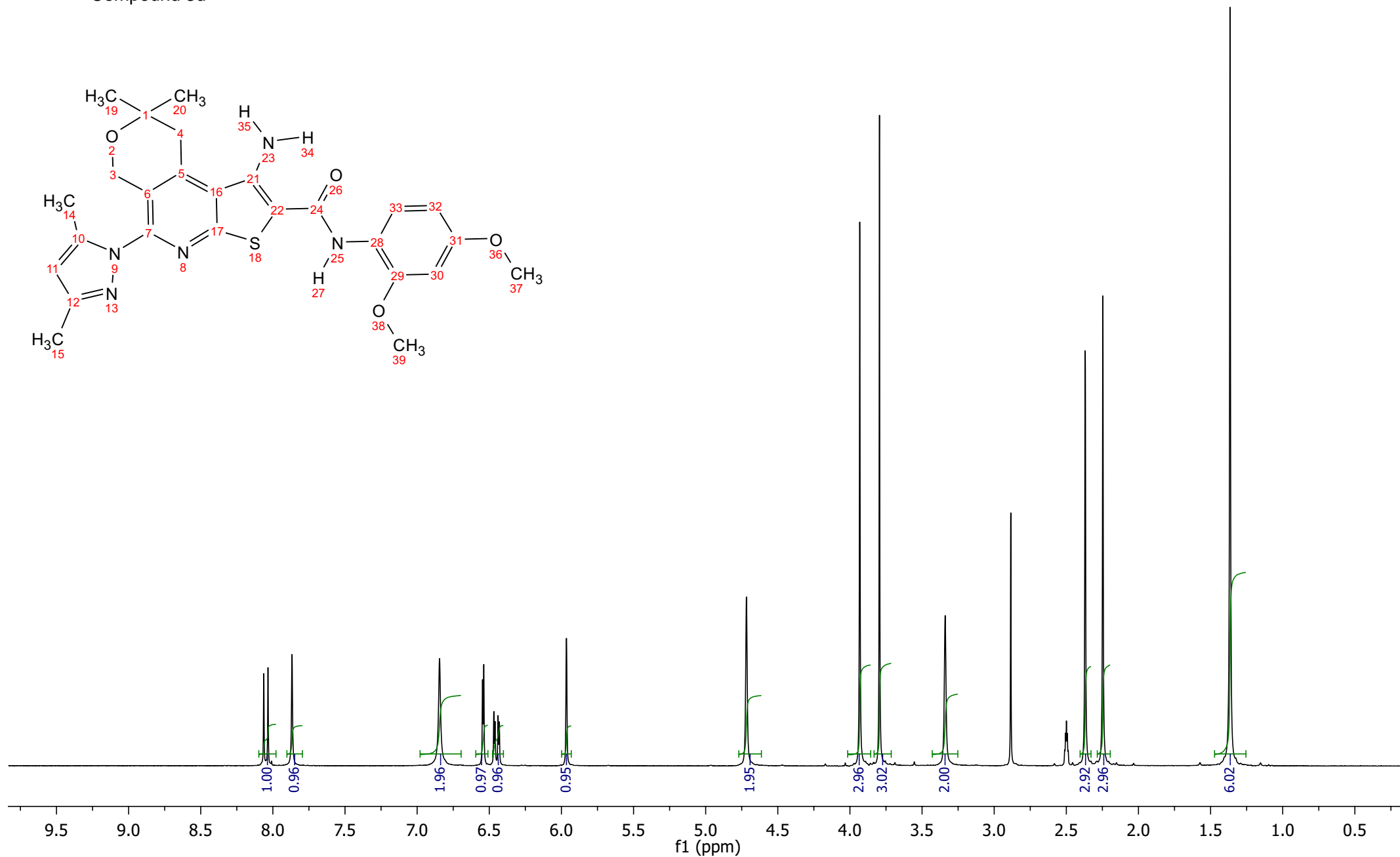

Supplement: Supplementary file 1 [file molecules-27-03380-s001.zip › comp.3d_H1.pdf]

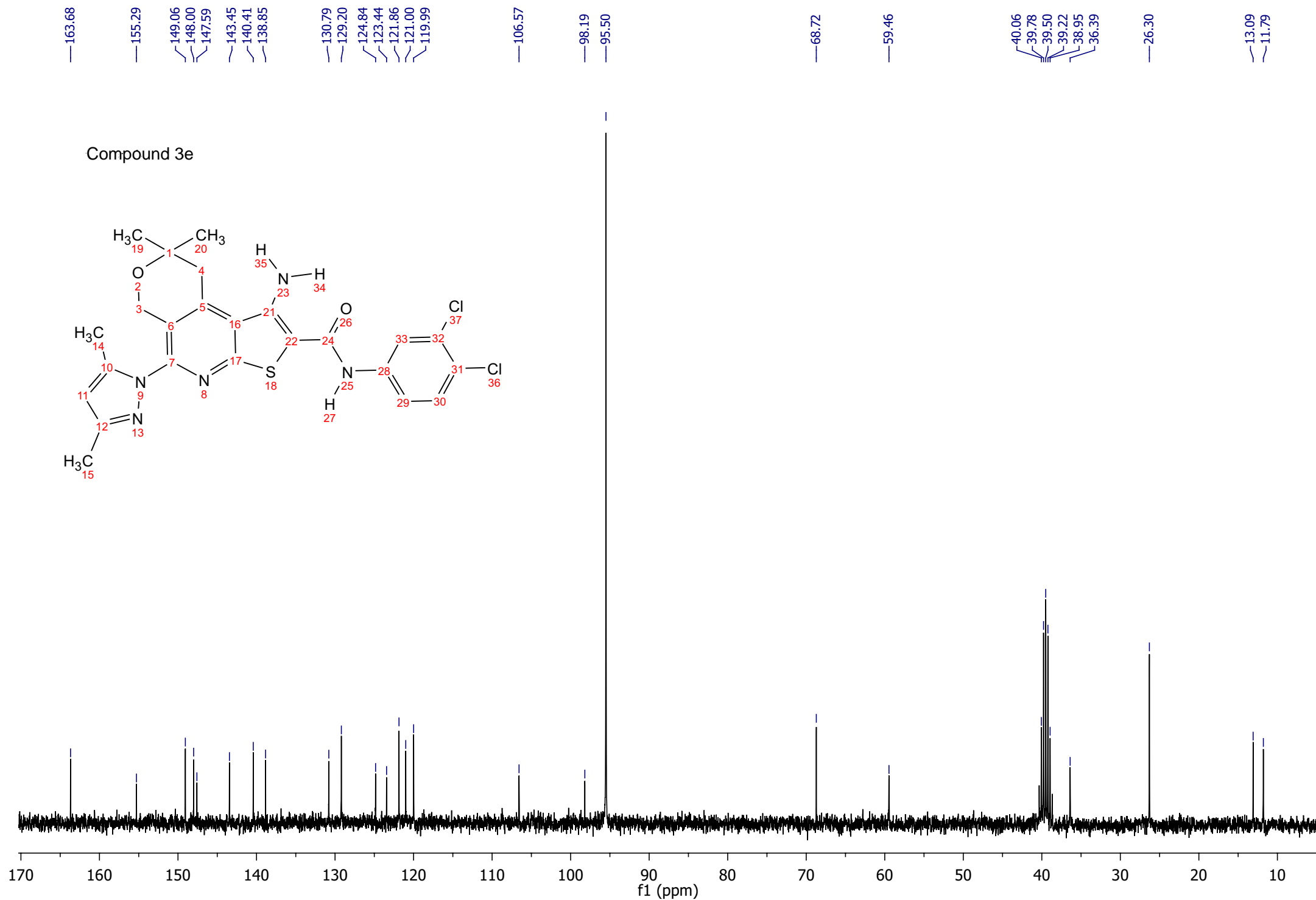

Supplement: Supplementary file 1 [file molecules-27-03380-s001.zip › comp.3e_C13.pdf]

Compound 3e

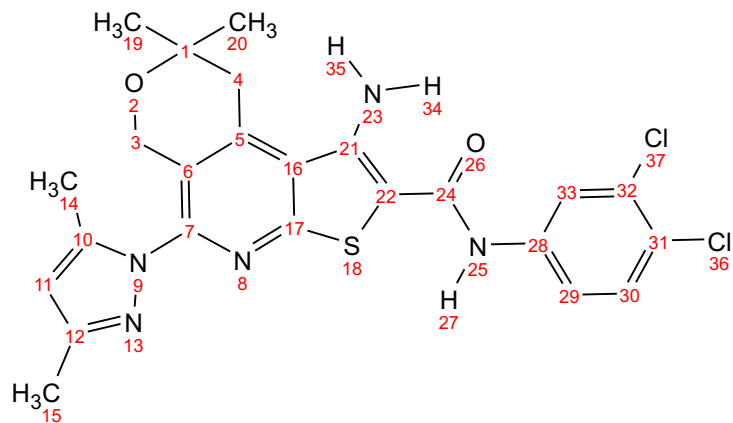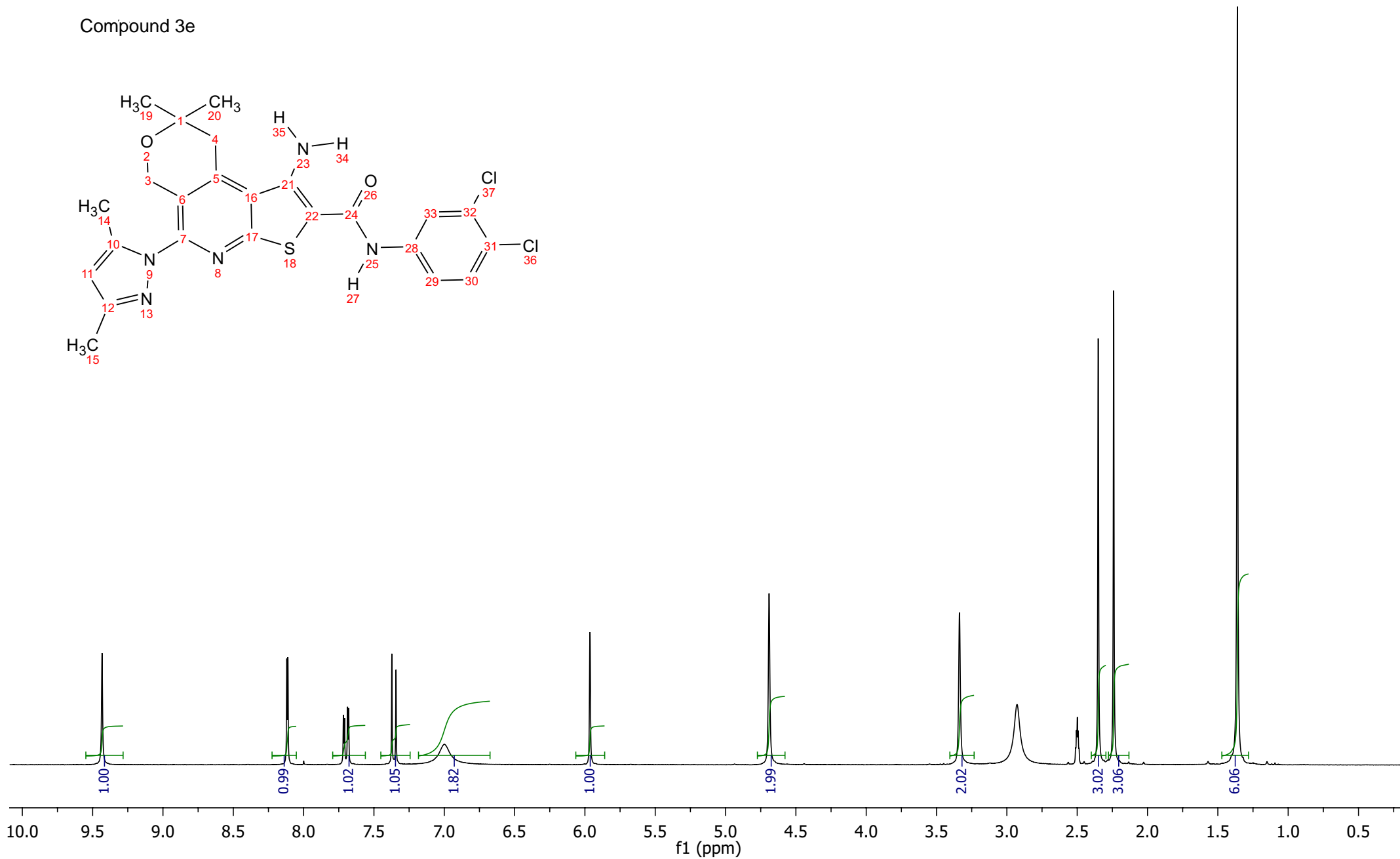

Supplement: Supplementary file 1 [file molecules-27-03380-s001.zip › comp.3e_H1.pdf]

Compound 3f

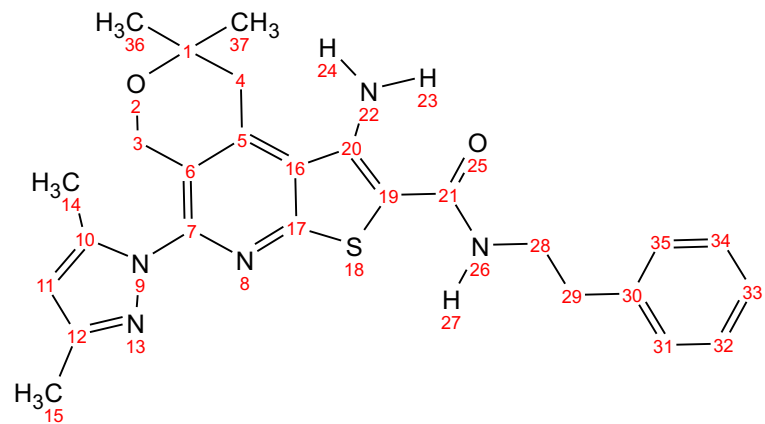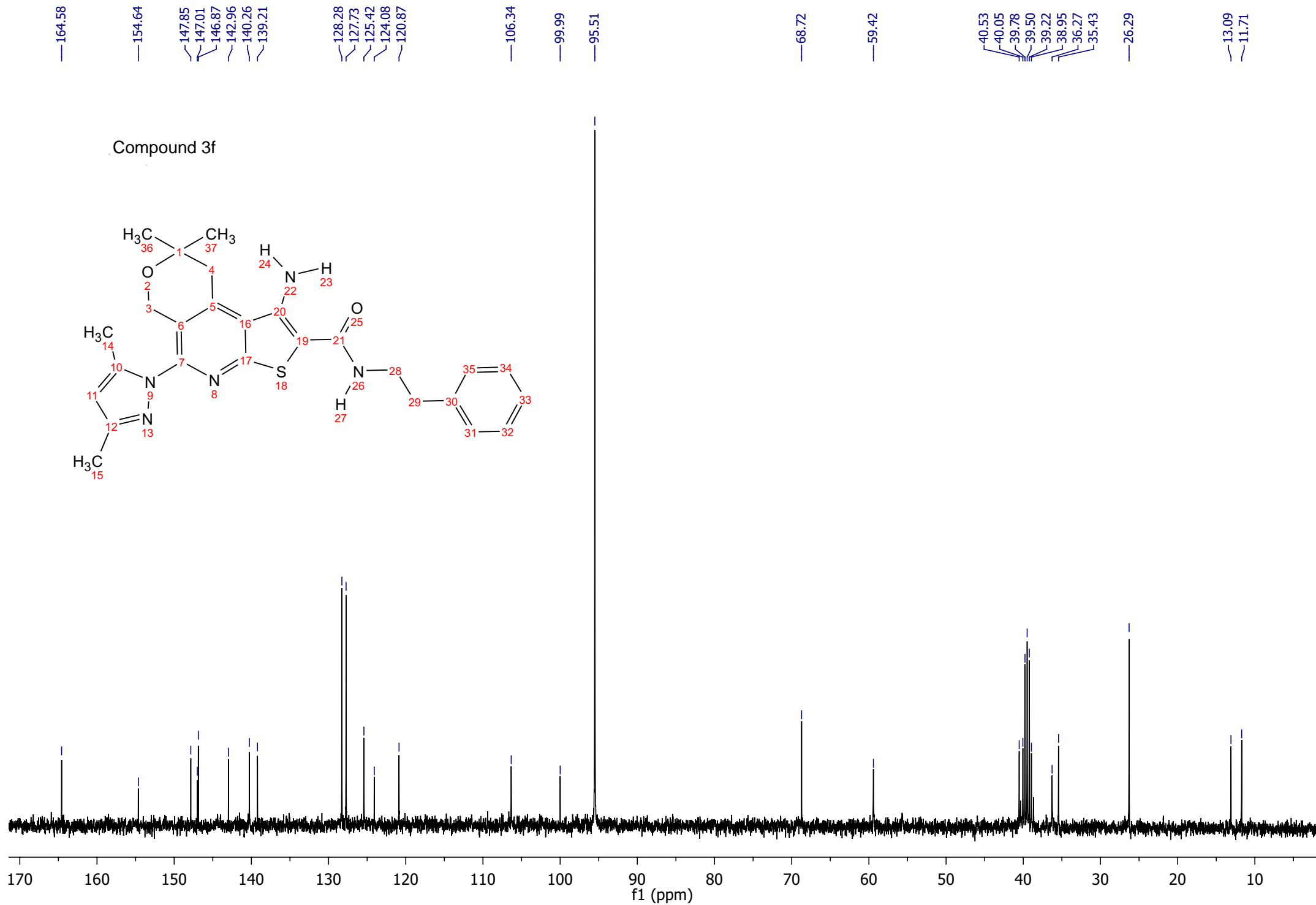

Supplement: Supplementary file 1 [file molecules-27-03380-s001.zip › comp.3f_C13.pdf]

Compound 3f

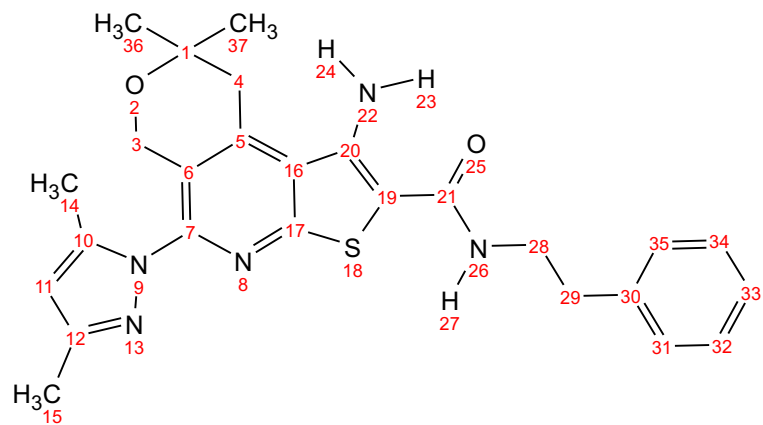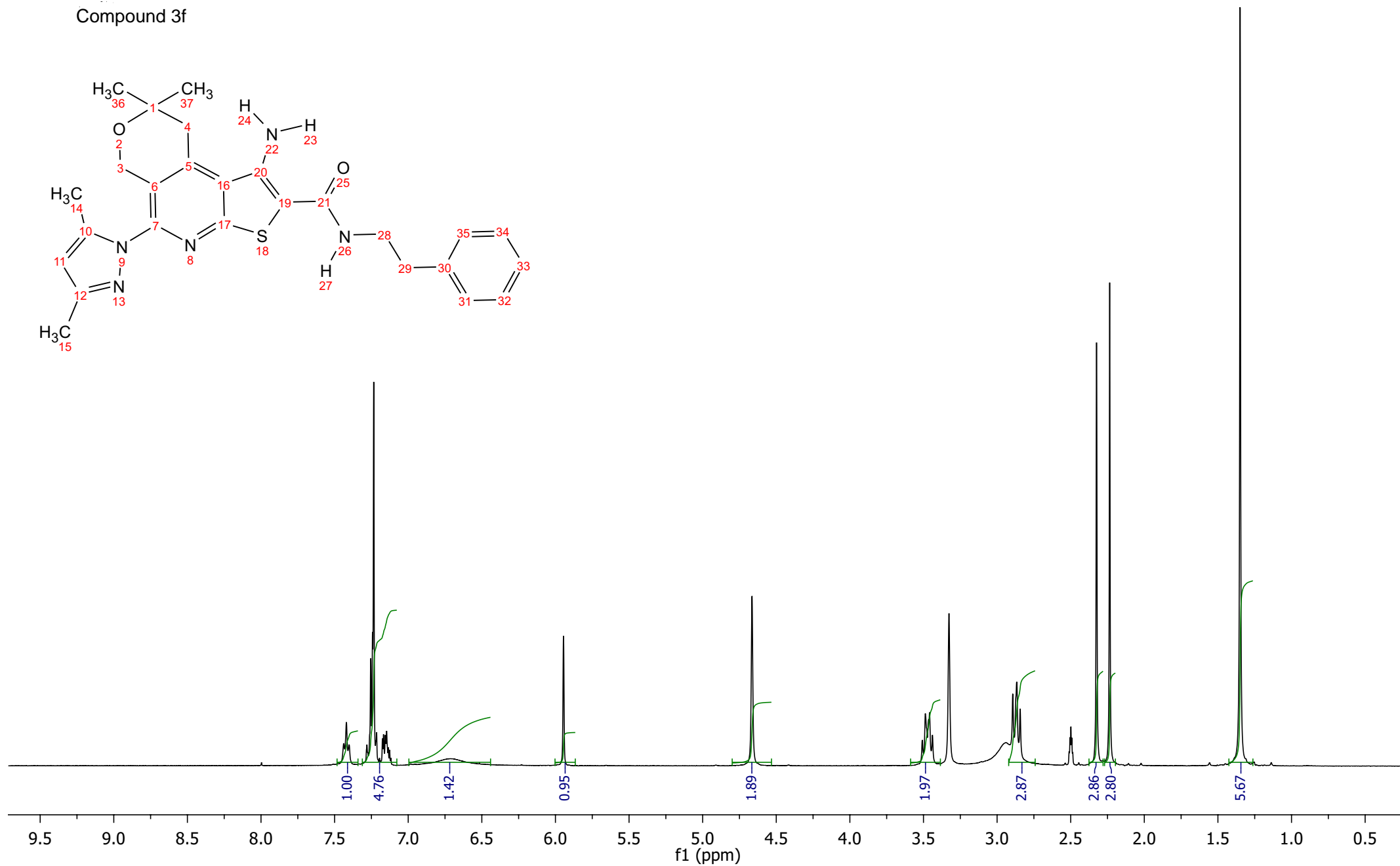

Supplement: Supplementary file 1 [file molecules-27-03380-s001.zip › comp.3f_H1.pdf]

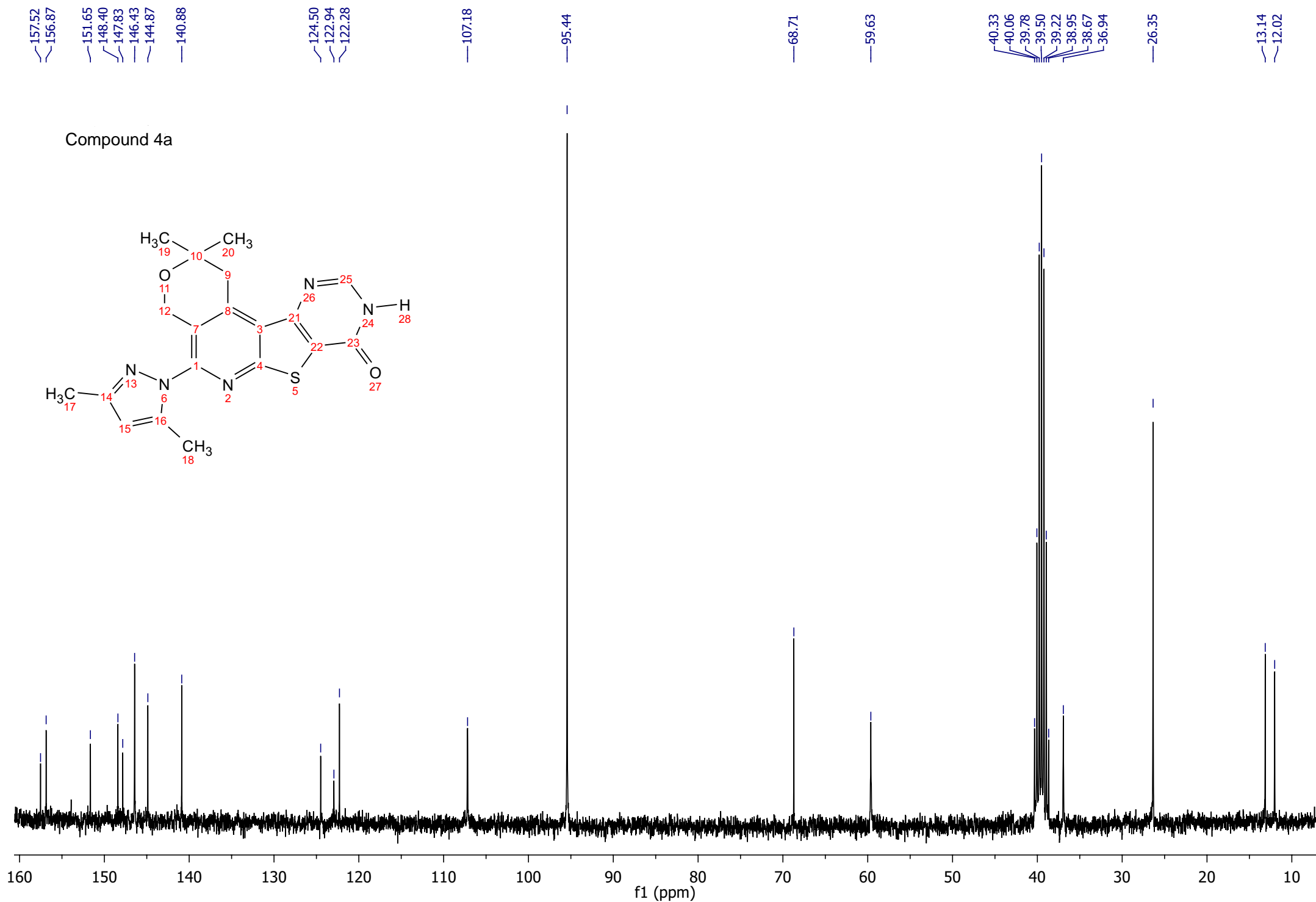

Supplement: Supplementary file 1 [file molecules-27-03380-s001.zip › comp.4a_C13.pdf]

Compound 4a

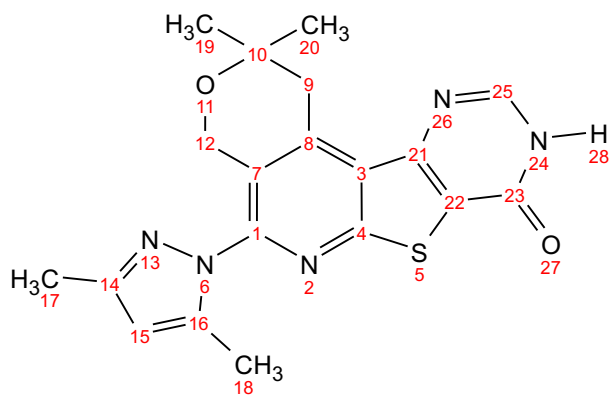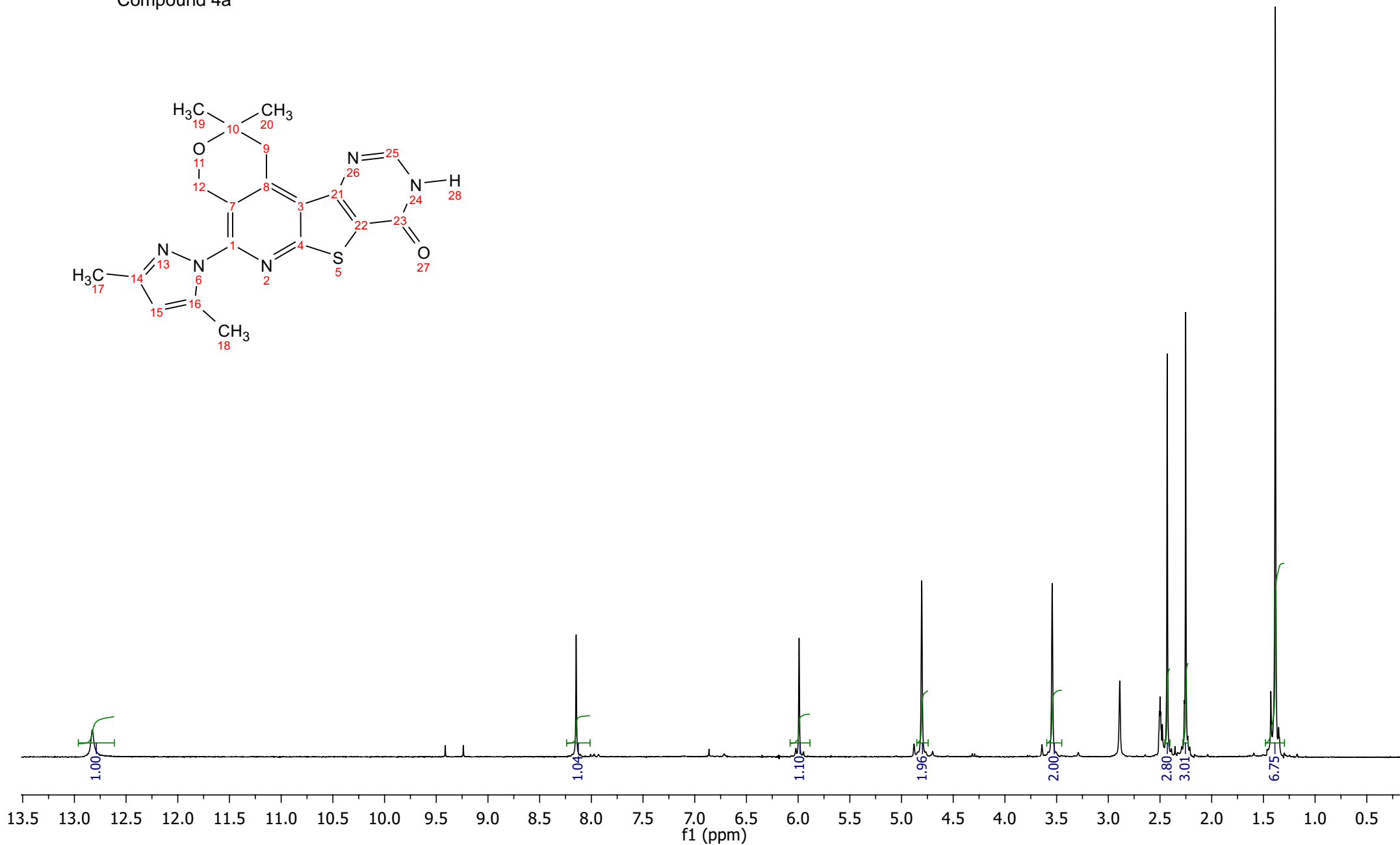

Supplement: Supplementary file 1 [file molecules-27-03380-s001.zip › comp.4a_H1.pdf]

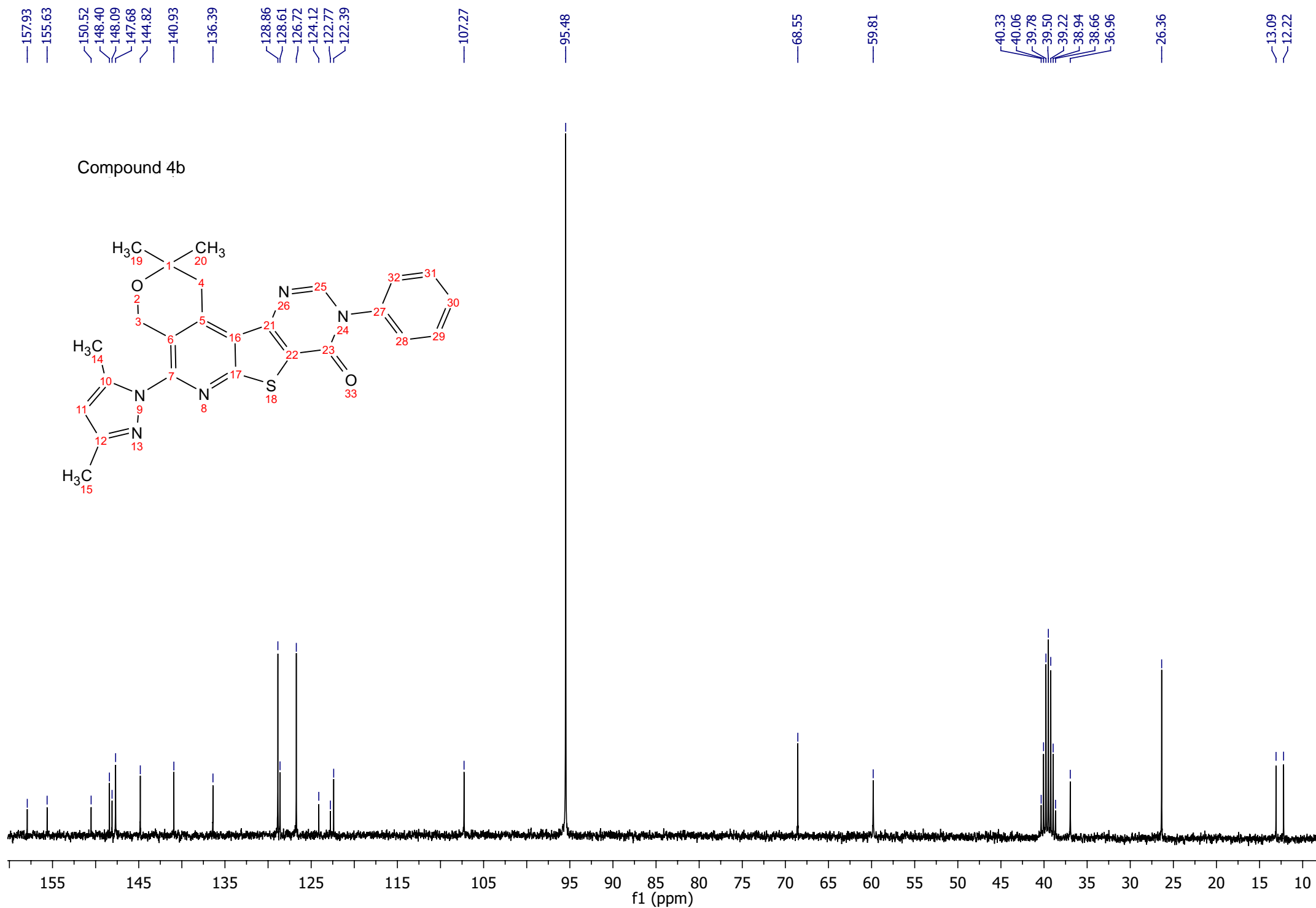

Supplement: Supplementary file 1 [file molecules-27-03380-s001.zip › comp.4b_C13.pdf]

Compound 4b

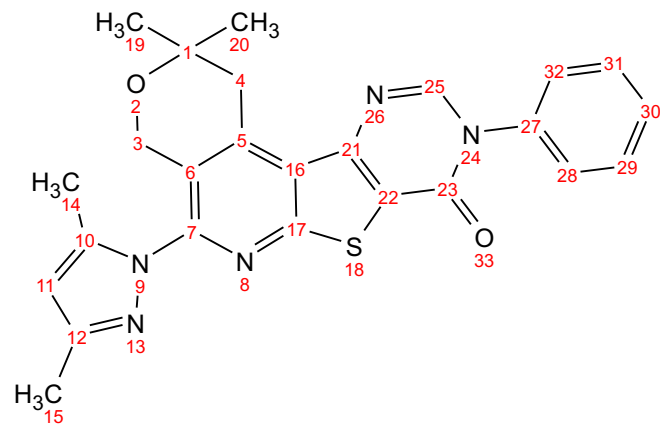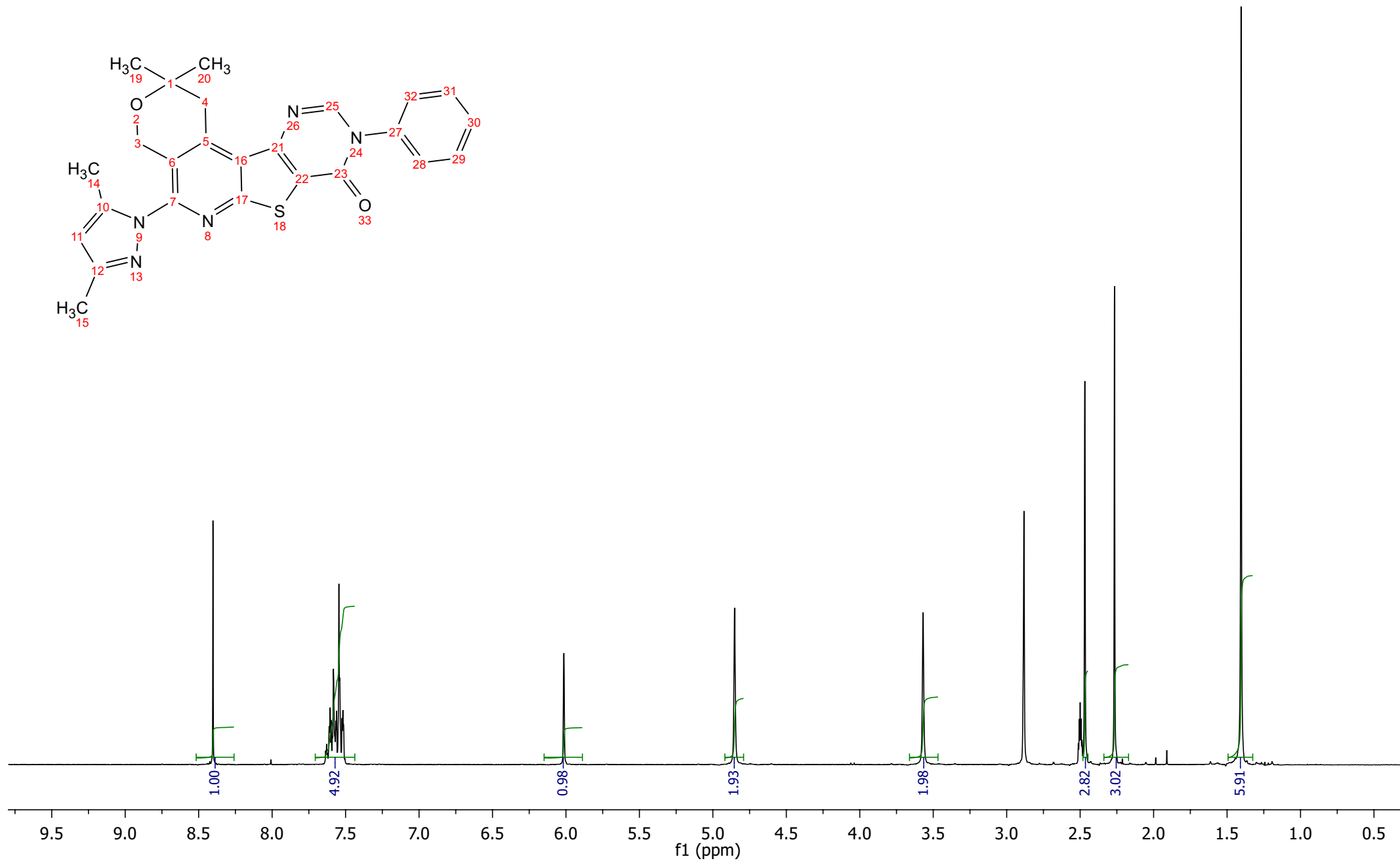

Supplement: Supplementary file 1 [file molecules-27-03380-s001.zip › comp.4b_H1.pdf]

Compound 4c

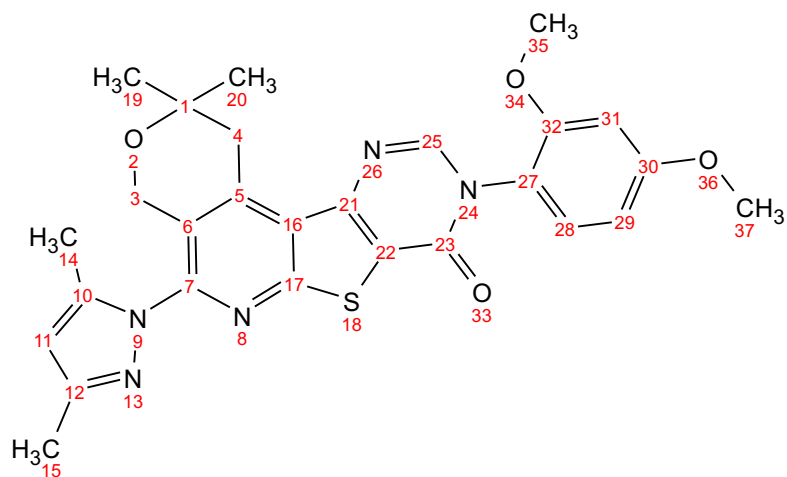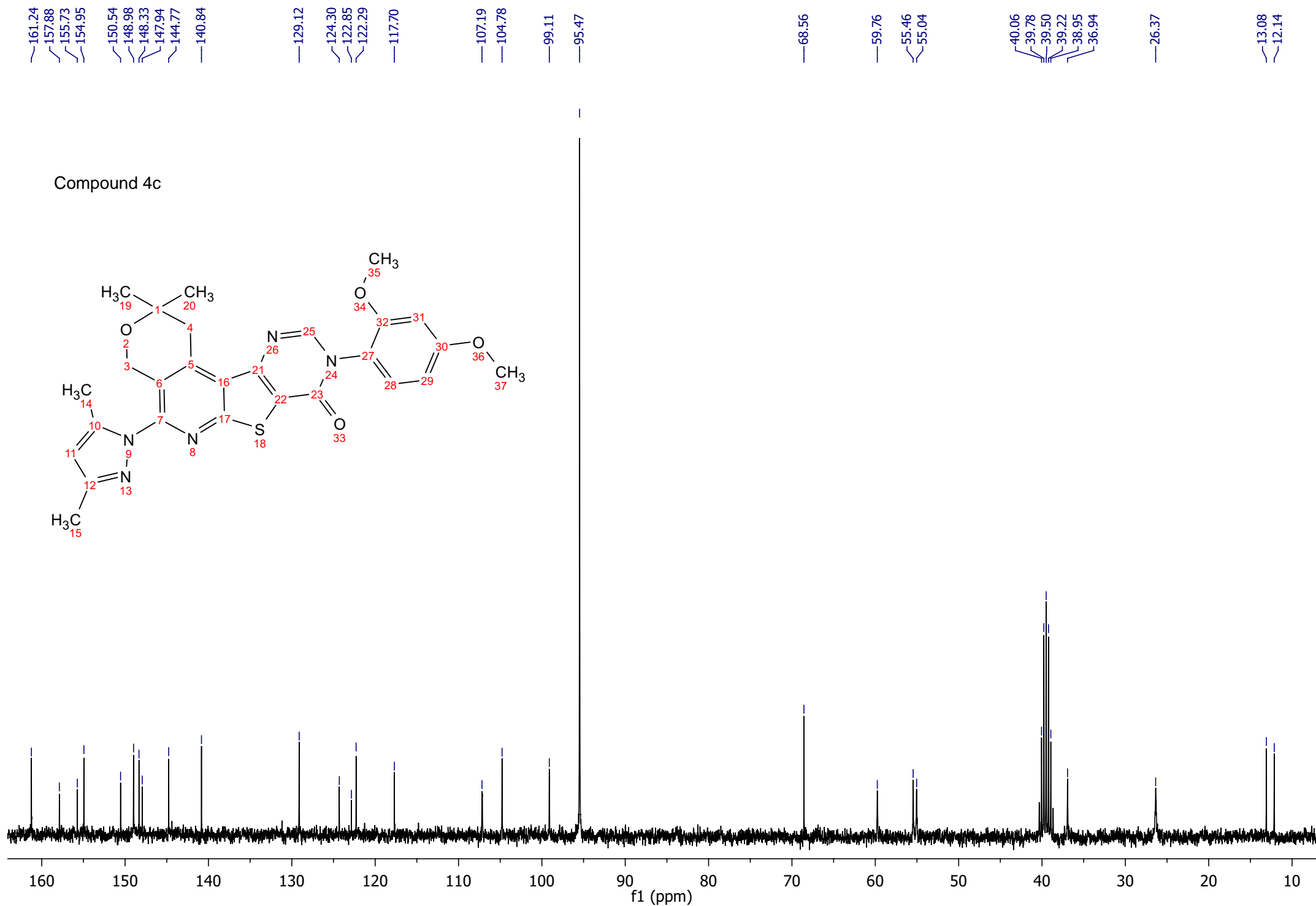

Supplement: Supplementary file 1 [file molecules-27-03380-s001.zip › comp.4c_C13.pdf]

Compound 4c

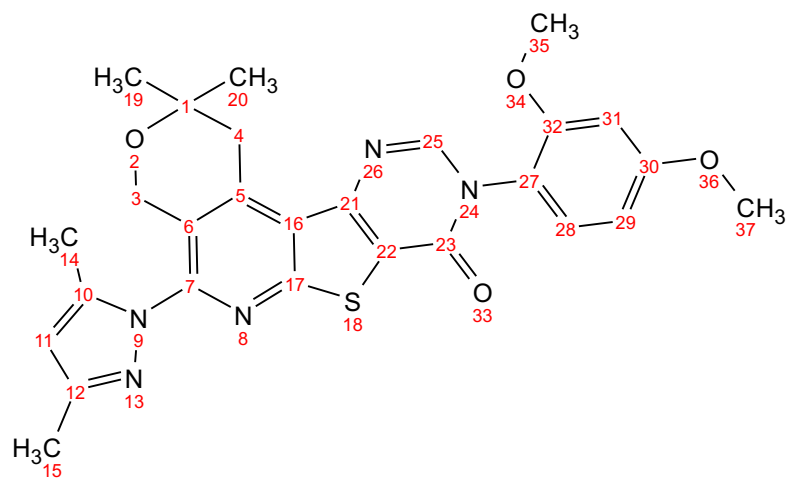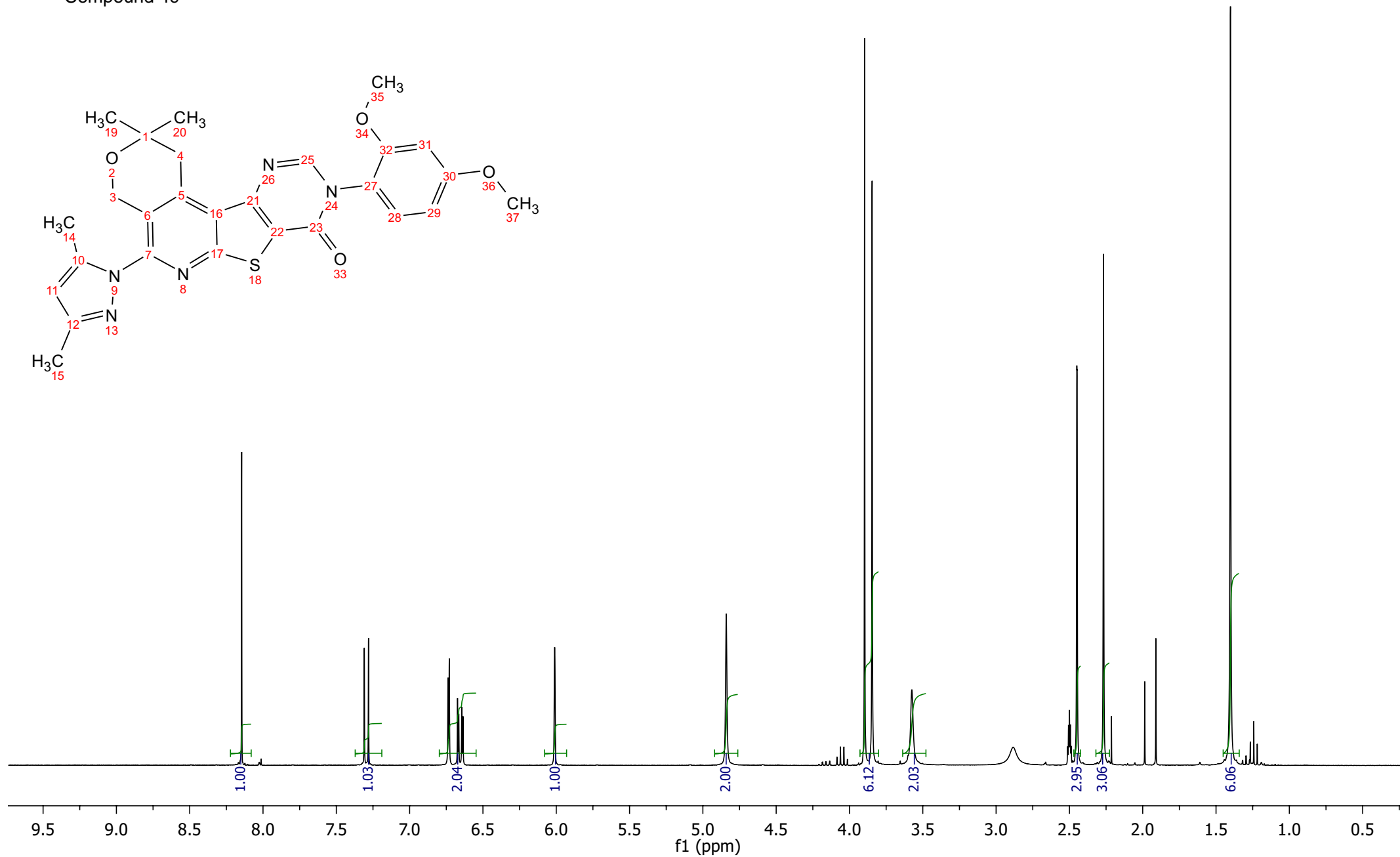

Supplement: Supplementary file 1 [file molecules-27-03380-s001.zip › comp.4c_H1.pdf]

Compound 4d

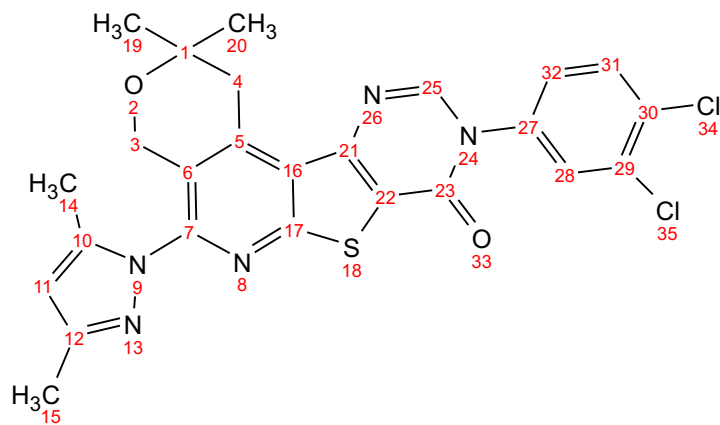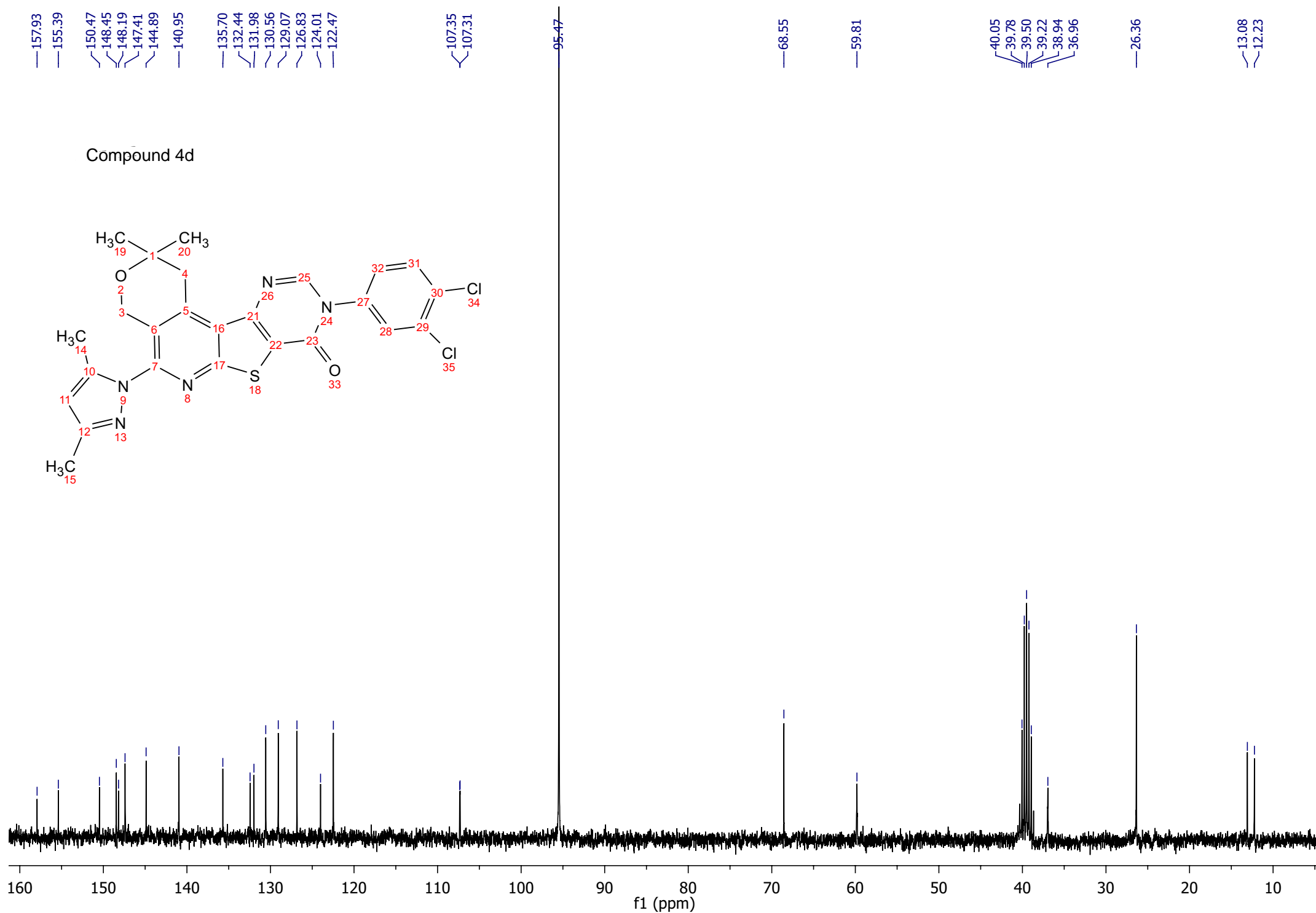

Supplement: Supplementary file 1 [file molecules-27-03380-s001.zip › comp.4d_C13.pdf]

Compound 4d

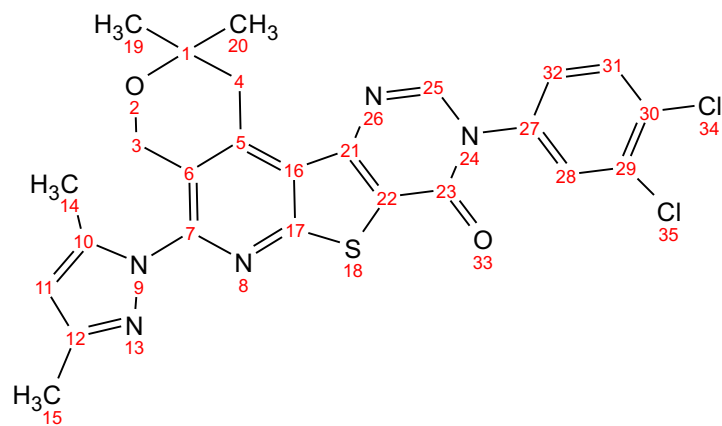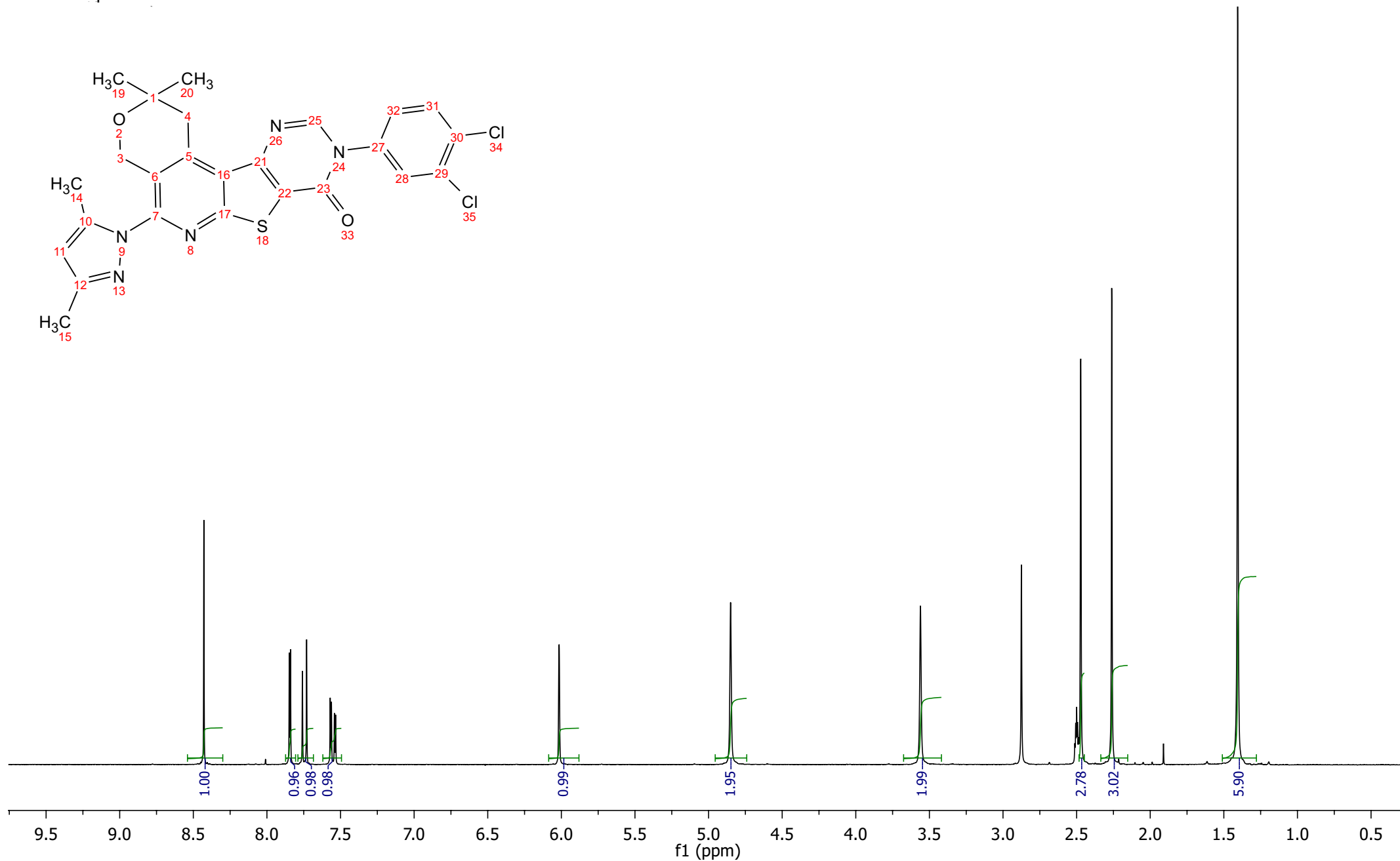

Supplement: Supplementary file 1 [file molecules-27-03380-s001.zip › comp.4d_H1.pdf]

Compound 4e

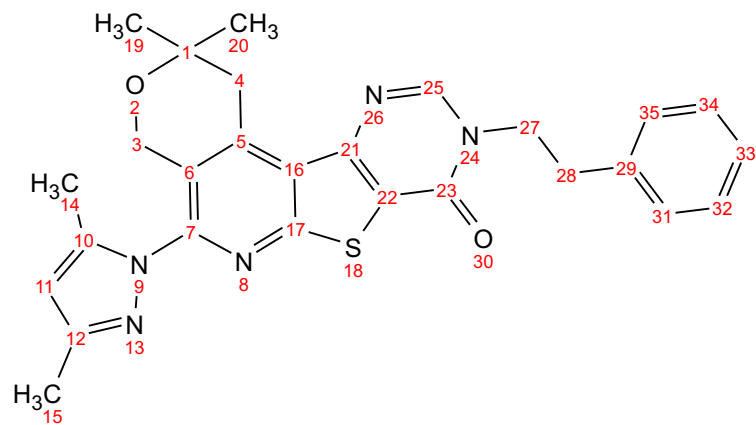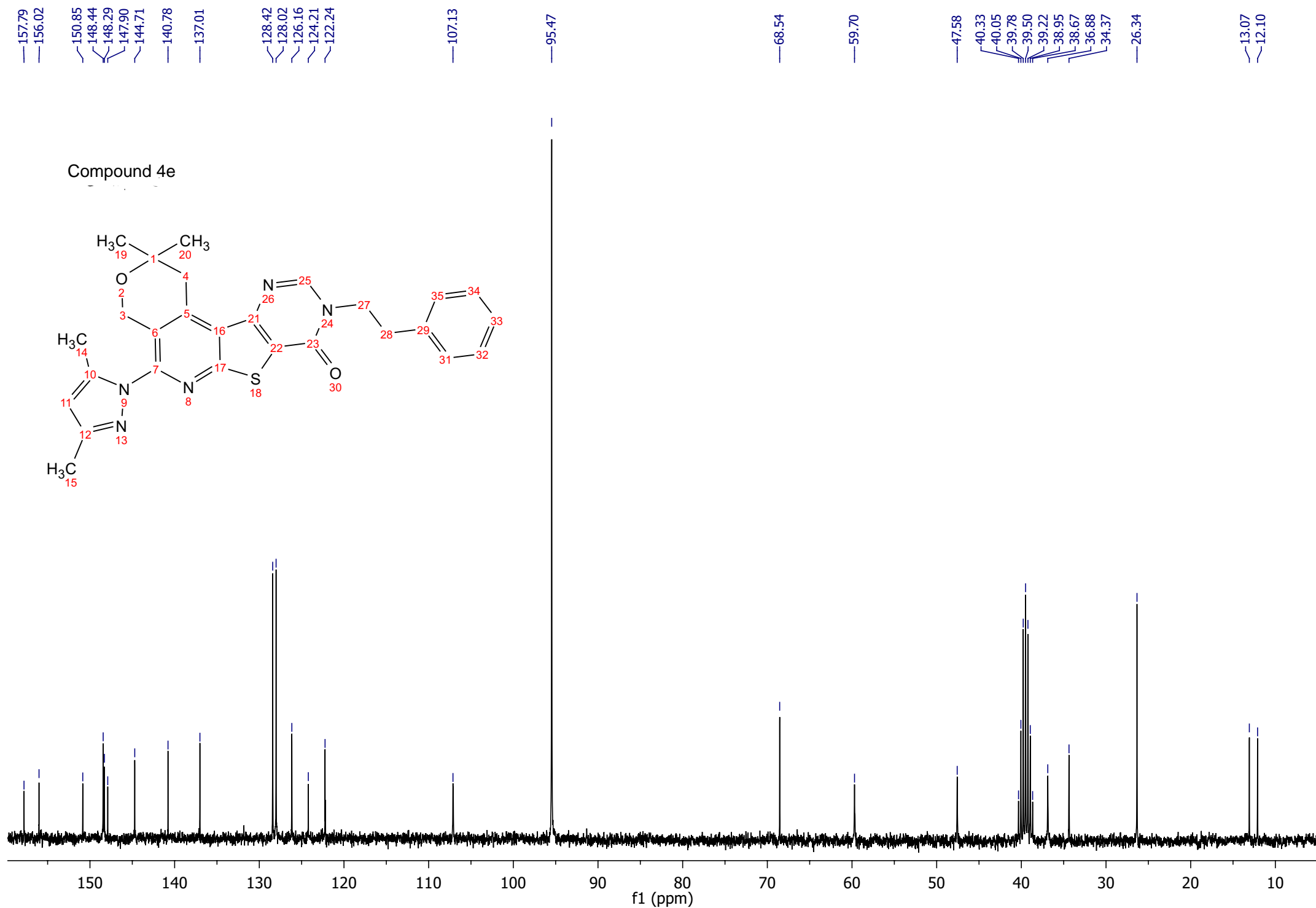

Supplement: Supplementary file 1 [file molecules-27-03380-s001.zip › comp.4e_C13.pdf]

Compound 4e

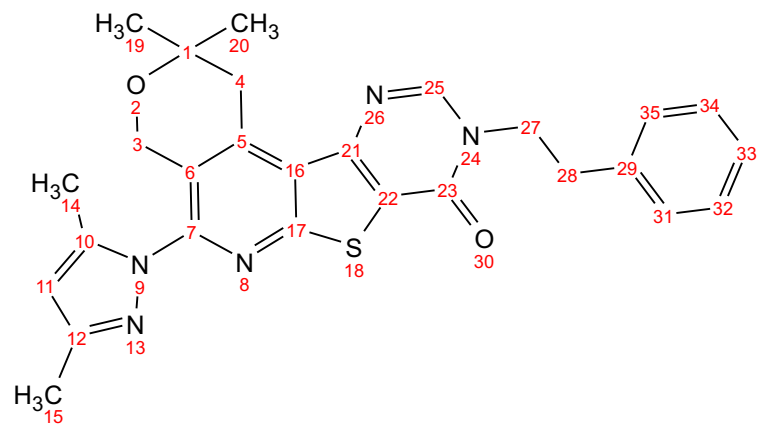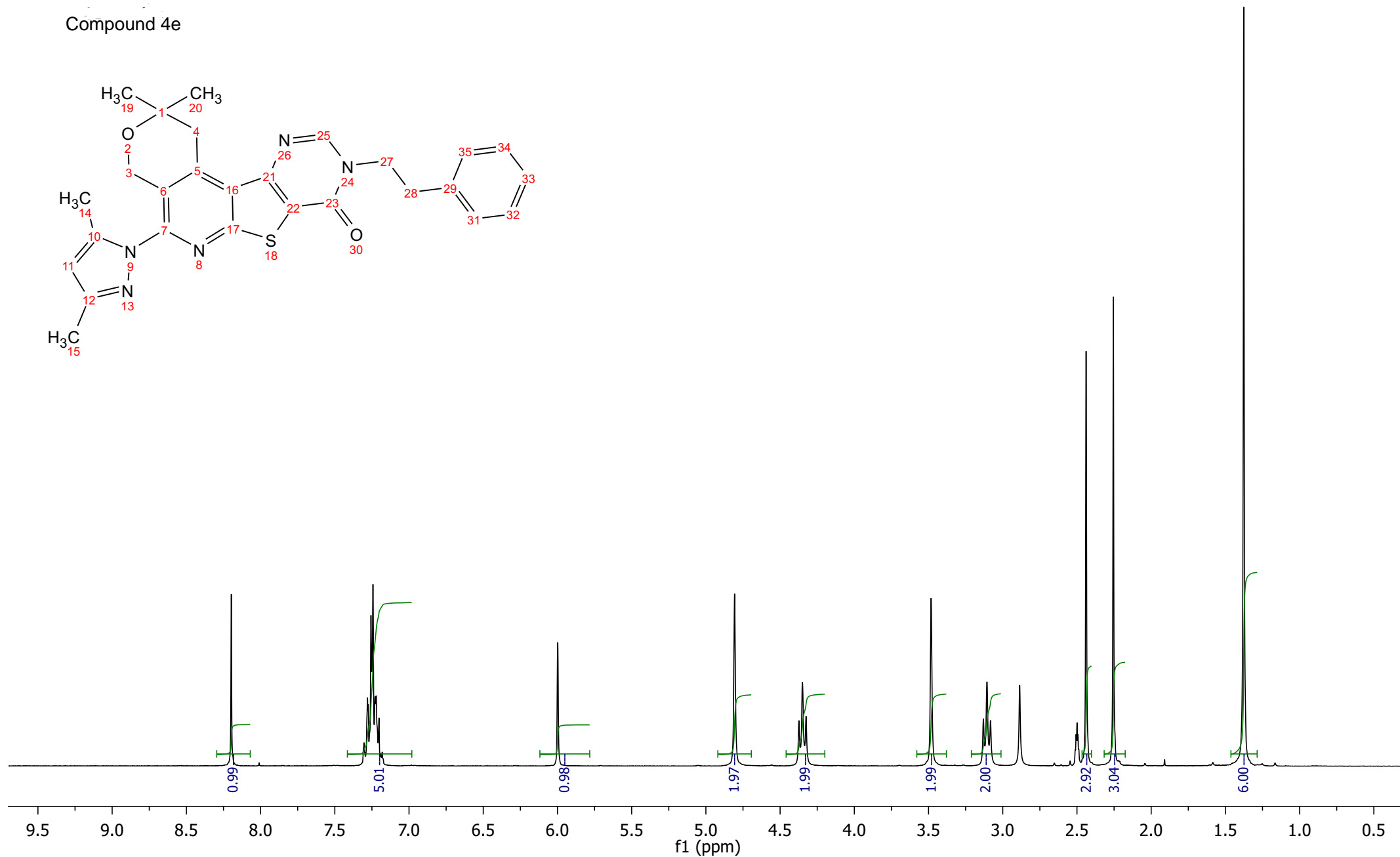

Supplement: Supplementary file 1 [file molecules-27-03380-s001.zip › comp.4e_H1.pdf]
